# Radiomics with artificial intelligence for precision medicine in radiation therapy

**DOI:** 10.1093/jrr/rry077

**Published:** 2018-09-22

**Authors:** Hidetaka Arimura, Mazen Soufi, Hidemi Kamezawa, Kenta Ninomiya, Masahiro Yamada

**Affiliations:** 1 Division of Medical Quantum Science, Department of Health Sciences, Graduate School of Medical Sciences, Kyushu University, 3-1-1, Maidashi, Higashi-ku, Fukuoka, Japan; 2 Division of Information Science, Graduate School of Science and Technology, Nara Institute of Science and Technology, 8916-5, Takayama-cho, Ikoma, Nara, Japan; 3 Department of Radiological Technology, Faculty of Fukuoka Medical Technology, Teikyo University, 6-22, Misaki-machi, Omuta, Fukuoka, Japan

**Keywords:** radiomics, artificial intelligence, precision medicine, radiation therapy, medical images, cancer traits

## Abstract

Recently, the concept of radiomics has emerged from radiation oncology. It is a novel approach for solving the issues of precision medicine and how it can be performed, based on multimodality medical images that are non-invasive, fast and low in cost. Radiomics is the comprehensive analysis of massive numbers of medical images in order to extract a large number of phenotypic features (radiomic biomarkers) reflecting cancer traits, and it explores the associations between the features and patients’ prognoses in order to improve decision-making in precision medicine. Individual patients can be stratified into subtypes based on radiomic biomarkers that contain information about cancer traits that determine the patient’s prognosis. Machine-learning algorithms of AI are boosting the powers of radiomics for prediction of prognoses or factors associated with treatment strategies, such as survival time, recurrence, adverse events, and subtypes. Therefore, radiomic approaches, in combination with AI, may potentially enable practical use of precision medicine in radiation therapy by predicting outcomes and toxicity for individual patients.

## INTRODUCTION

Artificial intelligence (AI) is based on computational algorithms or systems that can accurately perform high inference from a huge amount of knowledge data [1]. AI was named by John McCarthy *et al.* at a workshop at Dartmouth College in 1956, but the first and second booms of AI did not instill it either into daily life or into medicine. The current AI boom is the third wave since 2000. Hinton’s group made a breakthrough that almost halved the error rate for object recognition, using a convolutional neural network (CNN) or deep learning (DL), which is an advanced machine-learning technology [2], at the ImageNet Large Scale Visual Recognition Competition 2012 [3]. This event promoted the rapid adoption of DL by the computer vision community. DL architectures are composed of a cascade of multiple (deep) layers of non-linear processing units for learning datasets of input and output images and for extracting image features [3]. Since DL has a greater ability to recognize objects such as verbal and visual object patterns than conventional methods have, the applications of DL have been explored for segmentation of target regions in radiation therapy [4–7].

At around the same time as the third large wave of AI (after 2012), the idea of radiomics emerged from radiation oncology [8, 9] in the form of a novel approach for solving the issues of precision medicine, and how it can be performed based on multimodality medical images in a non-invasive (without biopsy), fast (fast scanning) and low-cost way (no additional examination cost). Radiomics is a new word derived from a combination of ‘radio’, which means radiological images (medical images in a broad sense), and ‘omics’ [10]. The omics are a number of fields of study (genomics, transcriptomics, proteomics and metabolomics) that improve our understanding of tumor biology and clinical management of cancer by comprehensively analyzing the massive amount of information become available on the genome, transcriptome, proteome and metabolome [11]. Precision medicine is a treatment strategy for making decisions about a molecularly targeted agent according to genetic mutations, rather than affected organs. Radiomics is a field that comprehensively analyzes massive numbers of medical images in order to extract a large number of phenotypic features (radiomic biomarkers) reflecting cancer traits, and explores the associations between the features and patients’ prognoses to improve decision-making in precision medicine [12]. Phenotypic features in medical images, which are routinely acquired in clinical practice, can result from the expression of the genotype (the organism’s genetic codes). The medical images are thought to include the internal information (e.g. anatomical, physiological and pathological information) in patients’ specific regions. Radiomics is considered a ‘specialized AI’ for predicting patient prognoses based on medical images. This review paper considers the following issues:

1. Why is radiomics needed in precision medicine?
2. The potential of radiomics for avoiding undesirable complications caused by biopsy
3. The overall procedure of radiomic analysis
4. What radiomic features reflect
5. What mathematical models of image features represent
6. Stratification of individual patients into subtypes using radiomic biomarkers
7. Radiomcs with AI (machine learning)
8. Perspectives of radiomics in radiation therapy.

## WHY IS RADIOMICS NEEDED IN PRECISION MEDICINE?

**Fig. 1.**
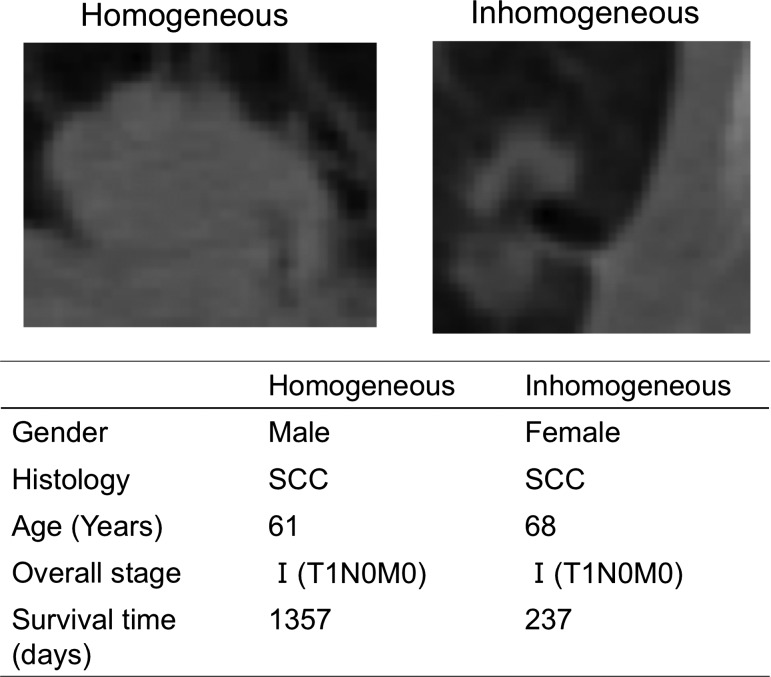
Two computed tomography (CT) images with the characteristics of two different tumors of lung cancer patients who each received radiation therapy. SCC, squamous cell carcinoma.

**Fig. 2.**
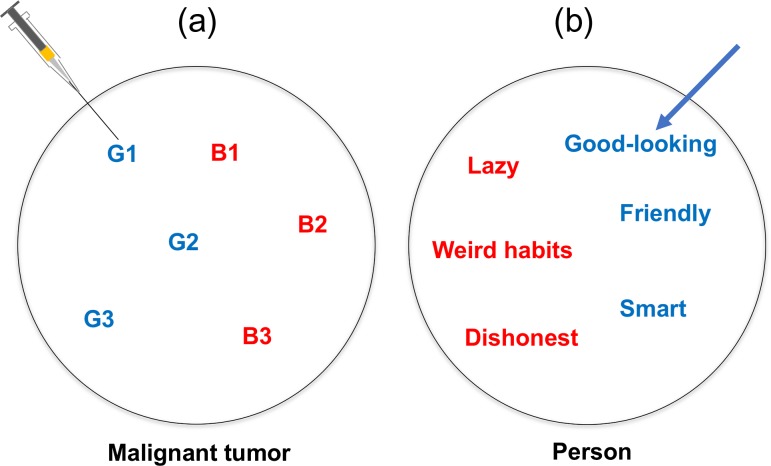
A gene-expression heterogeneity in the same tumor, and trait heterogeneity in the same person.

Figure 1 shows two computed tomography (CT) images showing the characteristics of two different tumors of lung cancer patients who received radiation therapy. They appear to differ in appearance in terms of intensity distribution and contour shapes from our subjective points of view, but they have similar histology and patient age. In fact, the two cancer patients had quite different survival times: 3.72 years for the homogeneous cancer (left) and 0.65 years for the inhomogeneous cancer (right), i.e. the patient on the left survived around five times as long as the one on the right. They could belong to different subtypes, from histological and genetic points of view. If the physician of the patient on the right could have predicted the survival time or prognosis prior to the treatment, different strategies might have been chosen. The idea that appropriate treatment strategies are selected according to subtypes is called ‘precision medicine’ [12]. In precision medicine, the subtypes of individual patients need to be identified; generally this is done using ‘wet’ biomarkers [13], i.e. biospecimen-derived biomarkers [14], such as genomic information derived from a part of a tumor obtained in a biopsy [12]. The Food and Drug Administration (FDA) in the USA defines a biomarker as a defined characteristic that is measured as an indicator of normal biological processes, pathogenic processes, or responses to an exposure or intervention, including therapeutic interventions [15]. Molecular, genomic, histologic, medical imaging, and physiologic characteristics can be considered as examples of biomarkers [16–18]. We now need to determine which biomarkers are more appropriate for the stratification of patients into subtypes that have different prognoses. Measurement of the biomarkers (other than medical imaging biomarkers) could require invasive biopsy or sampling of biospecimens, additional costs, and long examination tests. Furthermore, the biomarker information is limited if the biospecimens are taken from a part of an entire tumor using a single biopsy, because Gerlinger *et al.* [19] reported that gene-expression signatures of both good and poor prognoses were detected in different locations of the same tumor. Figure 2 describes gene-expression heterogeneity within one tumor, and trait heterogeneity within one person. The imaginary codes G1 to G3 and B1 to B3 represent gene-expression signatures of good and poor prognoses within the one malignant tumor, respectively, in Fig. 2a. A single biopsy of such a heterogeneous tumor could create a misleading estimation of prognosis, based on its genomic information. This situation is similar to that of trait heterogeneity in a person, as shown in Fig. 2b. If you liked a part of your partner, e.g. the positive side (good-looking, friendly, smart) (overestimation) before you lived together, you might be disappointed with the negative side (lazy, weird habits, dishonest) after you lived together.

The issues raised above are drawbacks of precision medicine. Aerts *et al.* [9] showed the prognostic powers of image features (statistical features and texture features) that have been derived solely from medical (CT) images of lung cancer patients treated with radiation therapy or radiochemotherapy, and the correlations of the image features with gene mutations. Their approach, based on medical images, could overcome the drawbacks of precision medicine, because medical images, which are acquired in a non-invasive, fast, and low-cost way, could obtain the entire information about cancer traits, such as intratumor heterogeneity. The signatures consisting of significant image features associated with patients’ prognoses can be considered ‘dry’ (imaging) biomarkers, and can extensively characterize cancer traits. Many imaging biomarkers based on radiomics have been explored by evaluating several end points to indicate the feasibility of radiomics to estimate overall survival and disease-free survival in radiation therapy [20].

## POTENTIAL OF RADIOMICS FOR AVOIDING UNDESIRABLE COMPLICATIONS CAUSED BY BIOPSY

Radiomics can be utilized to avoid undesirable complications caused by biopsy. Regarding brain tumors, craniotomy biopsy or stereotactic biopsy in brain tumors may cause some complications, such as intracerebral haematoma and hemiparesis, depending on tumor locations [21]. Some patients may refuse a needle biopsy to sample tissues from a suspected lung tumor due to concerns about a pneumothorax [22]. In these cases, physicians may decide the treatment policies for patients by reference to the radiomics results without the biopsy. The European Randomized study of Screening for Prostate Cancer (ERSPC) reported that prostate-specific antigen (PSA) screening decreased prostate cancer mortality, but that it increased substantial unnecessary biopsies, which resulted in undesirable complications such as fever, haematuria and haematospermia [23, 24]. In addition, according to Fukugai’s study [25], when using the original biopsy tumor grade rendered by nine different pathologists, the biopsy and prostatectomy results showed weaker agreement. Therefore, the information from radiomics can assist pathologists and radiologists by increasing the accuracy of biopsies or reducing unnecessary biopsies.

## OVERALL PROCEDURE FOR RADIOMIC ANALYSIS

**Fig. 3.**
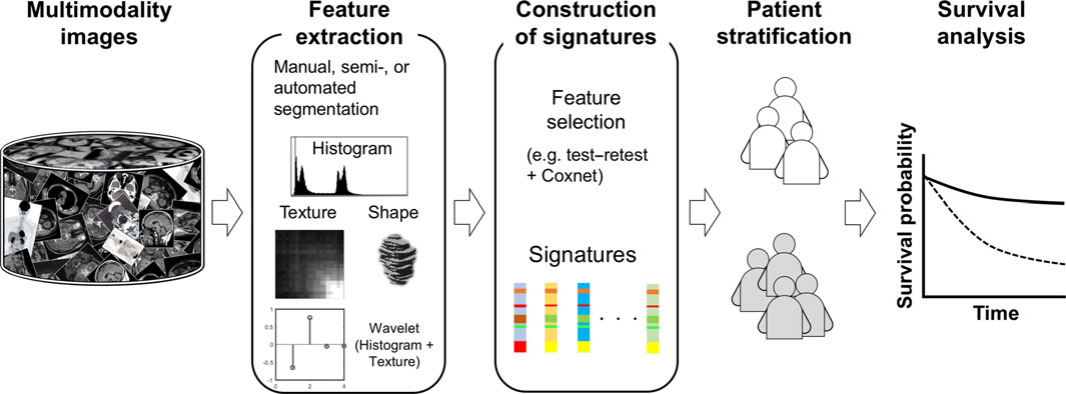
An overall procedure of radiomic analysis for discovering prognostic signatures that can predict patients’ prognoses.

Figure 3 illustrates an overall procedure of radiomic analysis for discovering prognostic signatures that can predict patients’ prognoses. The prognostic signature is a vector including significant features as elements. This procedure, shown in Fig. 3, is similar to what Aerts *et al.* [9] employed. First, a database including a large number of medical images [such as CT, magnetic resonance (MR) and positron emission tomography (PET) images for specific cancer patients who have received the same treatment] is prepared according to the requirements of radiomic analysis. Second, a large number of image features (e.g. >400), including texture features, are extracted from medical images after manual, semi- or automated segmentation. Third, radiomic features are selected based on their stability (demonstrated e.g. by test–retest [9]) as well as their contributions to the prognoses (e.g. the Coxnet algorithm [26]). Fourth, the patients are stratified into several subtypes of patients by using simple thresholding methods or clustering methods [2, 10]. In general, the threshold values are the medians of image features. Finally, the outcomes of the patients are predicted by performing survival analyses, in which significant features reflecting the prognoses are chosen as signatures. If there are statistically significant differences between the survival curves of two patient subtypes stratified by a radiomic feature, as shown in Fig. 3, the two patient subtypes could have different responses to a same-treatment approach. Therefore, the radiomic features may be associated with patients’ survival times.

## WHAT RADIOMIC FEATURES REFLECT

**Fig. 4.**
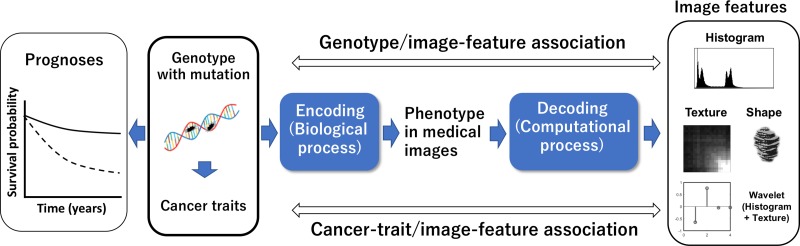
Radiomics assumptions about associations between prognoses and image features.

Figure 4 shows radiomic assumptions about associations between prognoses and image features. Genotypes with mutations could determine cancer traits that are associated with the prognoses of patients. On the pathway on the right in Fig. 4, the genotypes are believed to be encoded to the phenotypes expressed in medical images through biological processes. The image features may thus be derived by ‘decoding’ the phenotypes, i.e. medical images [9]. ‘Decoding’ medical images (phenotypes) refers to the computational extraction of image features from medical images using image processing and analysis techniques. Therefore, encoding followed by decoding means that the genotypes having associations with the cancer prognoses might be expressed by the image features, which are denoted as ‘radiomic features’. The image features could reflect cancer traits and prognoses. Furthermore, the authors believe that appropriate mathematical image features can model cancer hallmarks, which include six traits, i.e. sustaining proliferative signaling, evading growth suppressors, resisting cell death, enabling replicative immortality, inducing angiogenesis, and activating invasion and metastasis [27]. Possible associations between radiomic features and cancer hallmarks are introduced in Section ‘STRATIFICATION OF INDIVIDUAL PATIENTS INTO SUBTYPES USING RADIOMIC BIOMARKERS’.

## WHAT MATHEMATICAL MODELS OF IMAGE FEATURES REPRESENT

In general, the aim of image feature mathematical models is to characterize objects (lesions or anatomical structures in radiomics) that can be recognized in segmented regions, and whose image features are similar to those of objects in the same category, but different or as distinguishable as possible from those of objects in different categories [28]. Objective and quantitative image features would be expected to characterize abnormal lesions or malignant tumors, possibly reflecting the cancer hallmarks in radiomics.

Currently, there are three major types of mathematical feature models, i.e. shape, statistical, and texture features, that are utilized in the radiomics field. Shape features are surface area, surface-area-to-volume ratio, sphericity, spherical disproportion, compactness, and so on [29]. Statistical features obtained from a gray-level histogram represent overall heterogeneity without spatial information in terms of the gray levels within a tumor. Texture features derived from a gray-level co-occurrence matrix (GLCM) [30], gray-level run-length matrix (GLRLM) [31], neighborhood gray-tone difference matrix (NGTDM) [32] and gray-level size zone matrix (GLSZM) [33] represent the local spatial inhomogeneity in terms of gray levels within a tumor. The GLSZM features were employed for characterizing the inhomogeneity of cell nuclei [33].

To obtain the multiresolutional image features, including statistical and texture features, the wavelet transform has been applied for multiresolutionally decomposing multiscale local intensity variations (intratumor inhomogeneity) in an image into several low- and high-frequency components [34]. The multiresolutional decomposition was performed by using a ‘wavelet analysis filter bank’ approach [35] based on a 2D or 3D fast discrete wavelet transformation (fDWT) algorithm.

**Fig. 5.**
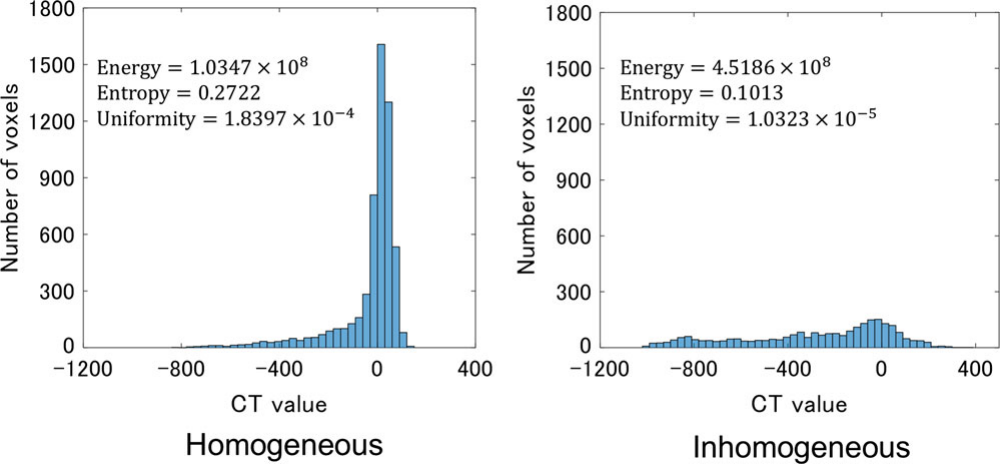
CT value (Hounsfield Unit) histograms of the homogeneous and inhomogeneous tumors (Fig. 1) for calculating statistical features.

Figure 5 shows CT value histograms of homogeneous and inhomogeneous tumors (Fig. 1) for calculating statistical features. The homogeneous and inhomogeneous tumors have narrow and broad CT value histograms, respectively. The broad histogram with the wide range of CT values indicates tissue inhomogeneity. The statistical features derived from the gray-level histograms are energy, entropy, kurtosis, maximum, mean, mean absolute difference, median, minimum, range, root mean square, skewness, standard deviation, uniformity, and variance, etc. [36].

**Fig. 6.**
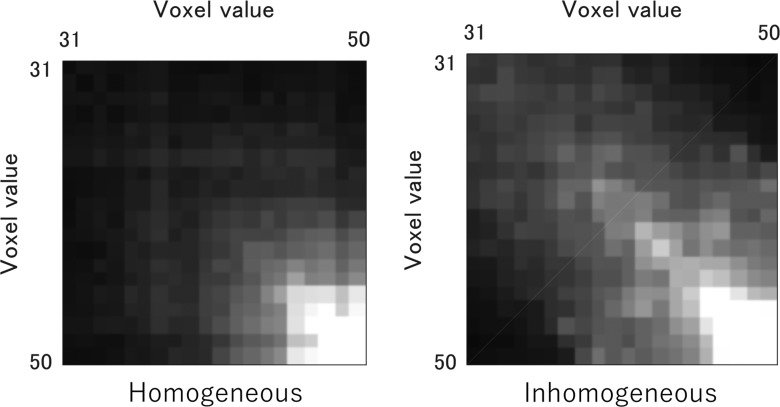
GLCMs of the homogeneous and inhomogeneous tumors (Fig. 1), which quantify the frequency of all possible combinations of grayscale values within neighboring voxels.

Figure 6 shows GLCMs of homogeneous and inhomogeneous tumors (Fig. 1), which quantify the frequency of all possible combinations of gray-scale values within neighboring voxels. The GLCMs are used for characterization of local tumor inhomogeneity by calculating many features, e.g. autocorrelation, contrast, correlation, cluster prominence, cluster shade, dissimilarity, energy, entropy, homogeneity, maximum probability, sum of squares variance, sum average, sum variance, sum entropy, difference variance, difference entropy, etc. [36]. Glioblastoma (GBM) cases have been classified by using GLCM, which characterized the GBM phenotypes [37].

## STRATIFICATION OF INDIVIDUAL PATIENTS INTO SUBTYPES USING RADIOMIC BIOMARKERS

In precision medicine with radiomics, individual patients should be stratified into subtypes using the radiomic biomarkers that contain information on cancer traits (mutation, angiogenesis, metastasis, immune escape) and determine patients’ prognoses (survival time, recurrence, toxicity), according to the various treatment strategies. Liu *et al.* [38] explored the association between CT-based image features and epidermal growth factor receptor (EGFR) mutation statuses with surgically resected peripheral lung adenocarcinomas. The highest area under the curve (AUC) of 0.709 was achieved in the prediction of the EGFR mutation using a logistic regression model for the combination of clinical factors and image features. The activation of EGFR mutations can be detected by use of image features for the stratification of patients in terms of their responses to tyrosine kinase inhibitors (TKIs) therapy for lung adenocarcinomas. Bak *et al.* [29] attempted to identify predictive imaging biomarkers that supported genomic alterations and clinical outcomes in patients with lung squamous cell carcinoma (SCC) using a radiomics approach. Mutational profiles for core signaling pathways of lung SCC were stratified into five subtypes: redox stress, apoptosis, proliferation, differentiation, and chromatin remodelers. The range of the gray-level histogram and the right lung volume were significantly associated with alternation of the apoptosis and proliferation pathways, respectively (*P* < 0.05).

Wang *et al.* [39] demonstrated that image features (morphological, gray-level, and statistical texture measures) of breast tumors and their surrounding parenchyma on dynamic contrast enhancement (DCE)-MRI could distinguish triple-negative breast cancers from other subtypes with higher accuracy (AUC of 0.878) than when considering the characteristics of the tumor alone, because triple-negative breast cancer would have responses to neither hormonal therapy nor anti-HER2 therapy. Ma *et al.* [40] investigated whether image features extracted from digital mammography images were associated with molecular subtypes of breast cancer. They showed the AUCs of 0.865 for triple-negative *vs* non-triple-negative, 0.784 for HER2-enriched *vs* non-HER2-enriched, and 0.752 for luminal *vs* non-luminal subtypes.

Yin *et al.* [41] sought to determine the associations between angiogenesis in renal cell carcinoma and imaging features from PET/MRI. Their study reported significant correlations between radiomic features and tumor microvascular density (MVD) (angiogenesis), and also demonstrated that spatiotemporal features extracted from DCE-MRI had higher correlations with MVD than the textural features extracted from Dixon sequences and ^18^F-fluoro-2-deoxdeoxyglucose PET (^18^F-FDG PET). Furthermore, PET/MRI, which took advantages of the combined metabolic and morphological information (radiomic features), had higher correlation with MVD than utilizing PET or MRI alone.

Yang *et al.* [42] developed and validated a radiomics-based nomogram to predict lymph node metastasis (LNM) in solid lung adenocarcinoma, because LNM of lung cancer was one of the significant factors that were relevant to survival and recurrence. In a validation cohort (*n* = 53), the AUC of the performance of LNM differentiation was 0.856. Shen *et al.* [43] constructed a radiomic nomogram for prediction of pre-operation LNM in esophageal cancer. The AUC was 0.771 in a validation cohort (*n* = 57).

Chen *et al.* [44] investigated the relationship between programmed cell death ligand 1 (PD-L1) expression and immunohistochemical (IHC) biomarkers or textural features of ^18^F-FDG PET in patients with head and neck cancer. The p16 (surrogate marker for human papillomavirus (HPV) involvement) and Ki-67 (proliferative marker) staining percentages detected using IHC and several ^18^F-FDG PET/CT–derived textural features can provide supplemental information to determine tumor PD-L1 expression. Subtypes with tumors with a higher PD-L1 expression may benefit from checkpoint inhibitors.

Cunliffe *et al.* [45] examined the correlation between the radiologist-defined severity of normal tissue damage following radiation therapy for lung cancer treatment and a set of CT-based texture features. Nineteen features that characterized the severity of radiologic changes from pre-therapy scans were identified. Furthermore, the same group assessed the relationship between radiation dose and changes in a set of mathematical gray-level– and texture-based features and the ability of image features to identify patients who would develop radiation pneumonitis (RP) [46]. This study demonstrated that radiomic features could discriminate between patient subtypes with and without RP.

## RADIOMCS WITH MACHINE LEARNING

A number of machine-learning algorithms [2] of AI technologies have been applied in radiomics for prediction or estimation of what will be associated with treatment strategies such as survival time, recurrence, adverse events, and tumor subtypes [47]. The uses of the machine learning, including feature selection methods, are comprehensive in many studies as described in this section, and the most appropriate machine-learning algorithms and feature selection methods are still unknown as we are yet to discover the combinations of significant features with the most prognostic powers.

Leger *et al.* [48] carried out a comparative study using 12 machine-learning algorithms combined with 11 feature selection methods, and the algorithm performances were assessed to predict loco-regional tumor control and overall survival for patients with head and neck squamous cell carcinoma in a multicenter cohort (*n* = 213). They found several combinations of machine-learning algorithms and feature selection methods that achieved similar results, e.g. random forest using maximally selected rank statistics had a concordance index of 0.71, and a Coxnet method with least absolute shrinkage and selection operator (LASSO) and elastic-net regularization based on boosting trees (BT) had a concordance index of 0.70 in combination with Spearman feature selection. Using the best performing models, patients were stratified into groups of low and high risk of recurrence. Parmar *et al.* [49] investigated 12 machine-learning approaches with 14 feature selection methods for radiomics-based prediction of 2-year survival of lung cancer patients treated with radical radiotherapy alone or with chemoradiation therapy. They identified that a Wilcoxon test–based feature selection method (stability = 0.84 ± 0.05, AUC = 0.65 ± 0.02) and a classification random forest method (relative standard deviation = 3.52%, AUC = 0.66 ± 0.03) had the greatest prognostic performances with relatively higher stability against data perturbation. Abdollahi *et al.* [50] proposed a radiomic analysis approach based on nine machine-learning tools and LASSO [2] for helping in the prediction of hearing loss induced by chemoradiation (cisplatin) therapy for head and neck cancer patients. The LASSO-penalized logistic modeling produced 10 predictive features with the highest performance, as indicated by an AUC of 0.885, for prediction of hearing loss. Shiradkar *et al.* [51] attempted to identify a radiomic signature derived from pretreatment biparametric MRI (bpMRI) that was predictive of prostate cancer biochemical recurrence after radical prostatectomy/radiotherapy/hormone therapy. The prediction accuracy using a machine learning of support vector machines (SVMs) with a feature selection of joint mutual information (JMI) was 0.84 in AUC. Gabryś *et al.* [52] investigated whether the machine-learning algorithms [2] (i.e. logistic regression with L1 penalty, logistic regression with L2 penalty, logistic regression with elastic net penalty, *k*-nearest neighbors, SVM, extra-trees, and gradient tree boosting) with dosiomic, radiomic, and demographic (age and sex) features allowed for xerostomia risk assessment in intensity-modulated radiation therapy (IMRT) for head and neck cancer (hypopharynx/larynx/nasopharynx/oropharynx/other). SVMs and extra-trees were the better performing classifiers compared with the others, whereas the algorithms based on logistic regression were the more appropriate choice for feature selection. Haga *et al.* [53] evaluated the potential application of radiomics for predicting the histology of early-stage non-small-cell lung cancer (NSCLC) patients, who underwent stereotactic body radiotherapy, by analyzing interobserver variability in tumor delineation. The average AUC for stratification of adenocarcinoma and squamous cell carcinoma subtypes was 0.725 using a naive Bayes model of machine learning.

## PERSPECTIVES OF RADIOMICS IN RADIATION THERAPY

Radiomics was able to predict patients’ prognoses based on extensive information about the tumors in a non-invasive, fast, and low-cost way, by stratifying patients into several subtypes such as EGFR and non-EGFR patients, using several imaging biomarkers. AI could be the key to successful radiomics for discovering biomarkers and determining patient stratification. We need to develop mathematical image-feature models of biomarkers to characterize cancer phenotypes or hallmarks and to select appropriate paths [54] in decision-making at each radiation treatment step (diagnosis, treatment planning, treatment execution, and follow-up). AI, including machine learning, may boost the prognostic powers of radiomics. Since many types of machine-learning software are open-sourced and easy-to-use for radiation oncology staff, this third boom in AI could be the practical phase of AI use in radiation oncology. Radiomic approaches may have one practical application in precision medicine by predicting outcomes and toxicity for individual patients in radiation therapy.
